# Expression of DKK1, HOXC6, and YKT6 Genes in Subjects With Oral Squamous Cell Carcinoma Residing in Central India: A Case-Control Study

**DOI:** 10.7759/cureus.98056

**Published:** 2025-11-28

**Authors:** Utsav Haldar, Niranjan Gopal, Kirankumar Prathipati, Amle Dnyanesh Balkrishna, Jyoti E John

**Affiliations:** 1 Biochemistry, All India Institute of Medical Sciences, Nagpur, Nagpur, IND; 2 Otolaryngology - Head and Neck Surgery, All India Institute of Medical Sciences, Nagpur, Nagpur, IND

**Keywords:** genetic oncology, oral cancer genetics, oral cavity cancer, oral oncology, risk factors oral cancer

## Abstract

Introduction

Oral squamous cell carcinoma (OSCC) is a predominant form of cancer affecting the head and neck region, accounting for the majority of cases in this category and posing a significant public health burden in Central India, where the incidence is among the highest in the country. Despite known associations with risk factors such as tobacco, alcohol, and areca nut use, the molecular mechanisms underlying OSCC progression and metastasis remain poorly understood. This study aimed to evaluate the gene expression profiles of *DKK1*, *HOXC6*, and *YKT6* in OSCC patients and explore their potential roles in tumour progression.

Methods

A case-control study was conducted at the All India Institute of Medical Sciences (AIIMS), Nagpur, between April 2023 and June 2024, involving 120 OSCC patients and 120 age- and gender-matched controls. Serum samples were collected for RNA extraction and RT-qPCR analysis of *DKK1*, *HOXC6*, and *YKT6*, using β-actin as a housekeeping gene. Expression differences were analysed using the 2^-ΔΔCt method and statistical tests, including t-tests and chi-square, with significance set at p<0.001.

Results

No significant differences in gene expression levels of *DKK1*, *HOXC6*, or *YKT6* were observed between OSCC cases and controls, or between subgroups with and without lymph node invasion. However, modifiable risk factors such as areca nut use (OR: 5.33), smoking (OR: 5.5), and alcohol consumption (OR: 9.85) were strongly associated with OSCC. The mean age of diagnosis was 57.5 years, with a male-to-female ratio of 2:1.

Conclusion

While *DKK1*, *HOXC6*, and *YKT6* did not demonstrate significant differential expression, the study highlights strong associations between OSCC and lifestyle-related risk factors. As the first gene expression study on OSCC in Central India, it underscores the need for larger, tissue-based studies to validate these findings and explore molecular targets for early diagnosis and immunotherapy.

## Introduction

The purpose of this study is to determine the roles of genes *DKK1*, *HOXC6*, and *YKT6* in biopsy-proven oral squamous cell carcinomas and to correlate tumour severity with gene expression levels.

Head and neck carcinomas (HNCs) are the seventh most common malignant neoplasm worldwide, with approximately 890,000 new cases and 450,000 mortalities annually [1]. Most of the HNCs arise from the squamous cells lining the mucosal epithelium and are further classified based on the anatomical location from which they are derived [2]. These include tumours affecting the oral cavity, nasopharynx, oropharynx, larynx, and hypopharynx. When combined, oral and pharyngeal cancer rank as the sixth most prevalent cancer globally [3].

It is estimated that oral cancer accounts for around 45% of cancer cases in India [4,5]. Particularly in central India, the age-adjusted rates (AAR) for oral cancer are 64.8% for males and 37.2% for females at 70 years of age, both statistics being the highest in the country [6]. In central India, 37% of all oral cavity neoplasms are malignant, while 63% are benign. The average age at presentation of malignant oral tumours is 51 years, and 23% of malignant oral tumours are first diagnosed in patients under 40 years of age [7]. Of all malignancies in the head and neck region, oral squamous cell cancer (OSCC) constitutes the majority at 90%. The heightened prevalence is primarily attributed to the extensive use of tobacco across India [8].

Metastasis, the spread of cancer cells from the primary tumour to distant sites, is a complex multistep process involving various genetic and molecular alterations. Gene expression profiling offers valuable insights into the dysregulated pathways and key drivers underlying OSCC metastasis [8]. In recent years, advancements in high-throughput technologies, such as microarray analysis and next-generation sequencing, have enabled researchers to comprehensively analyse gene expression patterns in OSCC. These studies have identified numerous genes and signalling pathways implicated in tumour progression, invasion, and metastasis. Dickkopf-related protein 1 (DKK1) emerges as a significant player in the context of OSCC metastasis [9]. In the context of invasion and metastasis, HOXC6 emerges as a key player implicated in the aggressive behaviour of cancer cells, including their propensity to invade surrounding tissues and disseminate to distant sites such as lymph nodes. Studies have consistently shown that dysregulated HOXC6 expression correlates with increased invasiveness and metastatic potential across different cancer types, including OSCC [10].

Mammalian genomes contain several *SNARE* genes, including *YKT6*, which is conserved across species. *YKT6* is involved in mediating membrane fusion events by forming complexes with other SNARE proteins, facilitating the docking and fusion of vesicles with target membranes. This process is very important for transporting cargo molecules between cellular compartments and for supporting cellular homeostasis. Recent studies have shed light on the diverse functions of *YKT6* beyond membrane fusion. *YKT6* has also been found to regulate intracellular trafficking pathways, including the endosomal-lysosomal system, Golgi apparatus, and autophagy. Moreover, *YKT6* is also involved in the regulation of cell migration, cytoskeletal dynamics, and cell signalling pathways involved in cell proliferation and survival [11]. Dysregulation of *YKT6* expression or function has been associated with various pathological conditions, including cancer.

## Materials and methods

This case-control study was conducted in the Department of Biochemistry in collaboration with the Department of Otorhinolaryngology at the All India Institute of Medical Sciences (AIIMS), Nagpur, spanning from April 2023 to June 2024 (approximately one year and three months). The study population included patients with biopsy-proven OSCC and healthy control subjects. Ethical approval was obtained from the Institutional Ethics Committee (IEC), and informed written consent was secured from all participants. The study adhered to the ethical principles outlined in the Declaration of Helsinki (1964) and its 2013 amendment. The sample size was calculated based on the assumption that *DKK1* relative gene expression had an odds ratio of 2.7 between cases and controls, with an expected expression rate of 68% among controls, a 5% alpha error, and a 95% confidence interval [12]. This yielded an estimated sample size of 106 cases and 106 controls. However, accounting for a 20% non-response rate and maintaining a 1:1 case-to-control ratio, the final estimated sample size was adjusted to 120 cases and 120 controls. *DKK1* was selected as the basis for the sample size calculation, as it yielded a larger sample size requirement than *HOXC6* and *YKT6* [13,14].

Inclusion and exclusion criteria for cases

Table 1 presents the inclusion and exclusion criteria for the cases included in the study.

**Table 1:** Inclusion and exclusion criteria for cases AJCC: American Joint Committee on Cancer

Inclusion Criteria	Exclusion Criteria
Biopsy-proven oral squamous cell cancer according to the modified AJCC classification (8^th^ edition) at any age, not on any treatment	Cases presenting with recurrence
	Cases coming for follow-up or revision surgery
	Cases coming after receiving treatment at another health facility
	Pre-malignant oral lesions in any age

Inclusion criteria for controls

Patients coming to the Outpatient Department for any complaint other than oral lesions. Controls were selected by a random sampling method over a period of one year. The control group was matched for age and gender with the cases. This was done by maintaining the gender ratio in both cases and controls. The percentage of subjects taken in the age groups <60 years and ≥60 years was also matched between cases and controls.

Data collection methods

All incident and prevalent patients from the Department of Otorhinolaryngology who met the inclusion criteria were invited to participate.

Relevant Clinical History

A detailed history of subjects, including demographic data, socioeconomic status, chief complaints (if any), history of presenting illness, personal history, family history, and treatment history, was documented. Furthermore, available medical records of the subjects were assessed for relevant medical information.

Methodology

Blood samples were collected in red-coloured 10 mL vacutainers from all cases and controls meeting the inclusion criteria. Following collection, the samples were centrifuged to separate the serum. The separated serum samples were stored at -80°C.

We investigated the transcriptional profiles of three genes (*DKK1*, *YKT6*, and *HOXC6*) in serum samples isolated from both control and test subjects. To achieve this, we have designed RT-qPCR assays tailored to these specific genes. The beta-actin gene (ActB) was used as the housekeeping gene for the purpose of internal control and validity of results. By analysing their expression levels, we aim to gain insights into their potential roles in the disease mechanisms under study and identify any significant differences between the control and test samples.

Following extraction, the RNA was stored at -80°C. This method offered a reliable and efficient protocol for RNA isolation, suitable for applications such as cDNA synthesis and RT-PCR.

Expression levels of the *DKK1*, *HOXC6*, and *YKT6* locus genes were quantified in all samples using RT-PCR. Quantitative RT-PCR was carried out using TaqMan PCR Master Mix (ThermoFisher Scientific, Mumbai, India) with uracil-N-glucosylase (UNG) probes that contain a reporter dye (FAM™ dye) linked to the 5`end of the probe, a non-fluorescent quencher (NFQ) at the 3` end of the probe and a minor groove binder moiety attached to the NFQ.

Nucleic acids have been found to have maximum absorbance at 260 nm. Historically, the ratio of absorbance at 260 nm to the absorbance at 280 nm has been used as a measure of purity in both DNA and RNA extractions. Thus, the 260/A280 and 260/A230 ratios indicate the purity of the RNA, with ideal values around 1.8-2.0 and 2.0-2.2, respectively. These ratios helped confirm that the RNA is of high quality and suitable for subsequent applications, such as RT-PCR.

The list of primers used for the gene expression studies is provided below (Table 2). Primers and oligos were designed according to standard RT-qPCR parameters using primer design tools (NCBI Blast, https://www.idtdna.com/scitools/Applications/RealTimePCR/default.aspx).

**Table 2:** Primer list used in RT-qPCR studied

Sr. No.	Name	Sequence	Length	Mature Amplicon Length
1	YKT6 F NM_001410874_1_c	CTACAATCCATTACCCAGCCC	21	104
2	YKT6 Probe (F-B) lcl_NM_001410874_1_c	TCAGTAGATACCAGAACCCACGAGAA GC	28	-
3	YKT6 R NM_001410874_1_c	TTTGGTCTCATCTAGTTCGGC	21	-
4	HOXC6 F NM_153693_5_cds_	TCACTTCAATCGCTACCTAACG	22	198
5	HXC6 Probe (F-B) NM_153693_5_cds_	ATCTGTCGCTCGGTCAGGCAAA	22	-
6	HOXC6 Rev NM_153693_5_cds_	TCCCGCTTTTCCTCTTTTCC	20	-
7	ACTB F BC014861_1_cds_A	TGAAGTGTGACGTGGACATC	20	106
8	ACTB Probe (F-B) BC014861_1_cds_A	CGCAAAGACCTGTACGCCAACAC	23	-
9	ACTB R BC014861_1_cds_A	GTGATCTCCTTCTGCATCCTG	21	-
10	DKK1 F NM_012242_4_cds_	TCCAACGCTATCAAGAACCTG	21	128
11	DKK1 Probe (F-B) NM_012242_4_cds_	CGCGCCGGGAATCCTGTACC	20	-
12	DKK1 R NM_012242_4_cds_	TGGTAGTTGTCAATGGTCTGG	21	-

The primer for the *HOXC6* gene was designed based on the work of Takahashi et al. (2003) [15].

**Figure 1:**
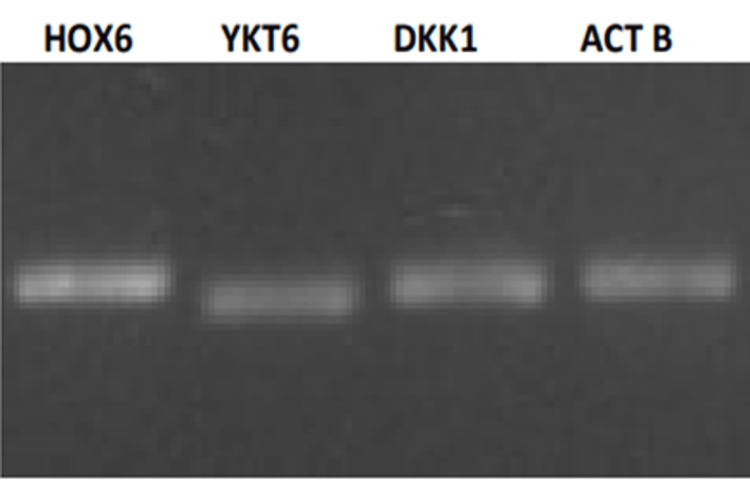
Agarose gel electrophoresis findings showing specific amplicon presence from the cDNA samples

After cDNA synthesis, we assessed primer specificity to confirm the suitability of the oligos for use in RT-qPCR. Specific amplification patterns from all four primers were observed over the agarose gel (Figure 1) in the pooled cDNA samples of control and test patients.

Statistical analysis

Data were recorded in a Microsoft Excel (Microsoft Corporation, Redmond, Washington) spreadsheet. The demographic and clinical characteristics of the patients were summarised using frequencies with percentages or means with standard deviations (SD). Analysis was performed using Microsoft Excel. For the age variable, the Shapiro-Wilk test was employed to determine if the data distribution was normal. Since the distribution was found to be not normal, the Mann-Whitney test was used to assess statistical differences between cases and controls. For other variables, chi-square tests were used to evaluate differences between cases and controls. We further subdivided the case population of our study into two groups: group I (cancer without any lymph node invasion) and group II (cancer with lymph node invasion and/or metastases). Statistical significance between the two groups was checked using the chi-square test. All assays were performed in triplicate, and the average threshold cycle (Ct) value was used to calculate relative gene expression. Log-transformed fold change was calculated using the 2-∆∆CT method and used to measure differential expression between the two groups [16]. The Shapiro-Wilk test was applied to determine whether the data set is normal, and Levene's test was used to assess whether the variances between cases and controls are equal. The data were normally distributed, and the variances were equal. The difference in gene expression between cases and controls was assessed using a two-sample t-test. Similarly, the difference in gene expression between grades I and II was also evaluated. Statistical analysis was considered significant at a p-value < 0.05.

## Results

Table 3 presents the demographic characteristics of the 240 subjects, including 120 OSCC patients and 120 age- and gender-matched controls. Regarding the age variable, the Shapiro-Wilk test was used to assess whether the data were normally distributed. The data were found to be normal; therefore, the Mann-Whitney test was used to assess any statistical significance between cases and controls. The mean age for cases was found to be 57.525 ± 11.63 years, and the mean age for controls was 58.725 ± 9.16 years. There were 81 males (67.5%) and 39 females (32.5%) in both cases and controls. The cases and controls were chosen in an age- and gender-matched manner, so no statistical significance was seen between them. Among the modifiable risk factors, it was observed that the number of subjects with addiction to areca nut chewing was significantly higher in cases compared to controls, with cases having 5.331 times more odds of areca nut exposure compared to controls. Similarly, the number of subjects with smoking addiction was also significantly higher among cases compared to controls, with cases having 5.5 times more odds of exposure to smoking than controls. There was also a significant difference between cases and controls with respect to the habit of alcohol consumption, with cases having 9.846 times more odds of being exposed to alcohol as compared to controls (Table 3).

**Table 3:** Demographic characteristics of the study population *: The age parameter is presented as mean ± SD. The Mann-Whitney test was used to assess any statistical significance between cases and controls. Cases and controls were chosen in an age-matched manner, so no statistical difference was found. **: Males and females are presented as percentages. Cases and controls were chosen in a gender-matched manner, so no statistical difference was found. ***: For the history of areca nut chewing and smoking and alcohol consumption, the data are presented as percentages. The chi-square test was used to assess for any statistical significance, with significance being considered at a p-value < 0.05.

Variables	Cases (n=120)	Controls (n=120)	p-value	Chi-Square Value	Odds Ratio	95% CI
Age in years (mean ± SD)*	57.52 ± 11.63	58.72 ± 9.16	-	-	-	-
Males (%)**	81 (67.5%)	81 (67.5%)	-	-	-	-
Females (%)**	39 (32.5%)	39 (32.5%)	-	-	-	-
History of areca nut chewing (%)***	97 (80.83%)	53 (44.16%)	<0.001*	34.42	5.331	2.993–9.492
History of smoking (%)***	80 (66.67%)	32 (26.67%)	<0.001*	38.57	5.5	3.163–9.560
History of alcohol consumption (%)***	91 (75.83%)	29 (24.16%)	<0.001*	64.06	9.846	5.464–17.744

We further divided the case population into two groups: Group I (cancer without lymph node invasion) and Group II (cancer with lymph node invasion and/or metastases). The chi-square test was used to evaluate statistical significance between these two groups. No statistically significant differences were observed (Table 4).

**Table 4:** Distribution of cases based on the severity of cancer across different variables Out of 120 cases, 48 (40%) were classified in group I (cancer with no lymph node involvement) and 72 (60%) were classified in group II (cancer with lymph node involvement and/or metastases). Data are presented as percentages. The chi-square test was employed to evaluate statistical significance, with a p-value considered significant if less than 0.05.

Variables	Group I (n=48)	Group II (n=72)	Chi-Square Value	p-value
Males (%)	34 (41.97%)	47 (58.03%)	0.405	0.525
Females (%)	14 (35.89%)	25 (64.11%)		
History of areca nut chewing (%)	39 (40.2%)	58 (59.8%)	0.009	0.925
History of smoking (%)	32 (40%)	48 (60%)	0.00	1.000
History of alcohol consumption (%)	38 (41.75%)	53 (58.25%)	0.485	0.486

Relative gene expression of target genes

Representative expression profiles of the transcripts and the Ct values observed in a plate setup format clearly showed high PCR efficiency and consistent Ct values across replicates and various samples. For RT-PCR, 24 samples were used (12 cases and 12 controls). The 24 samples were run in triplicate. The mean of the log-transformed values for each of the three genes was used for all statistical analyses.

We assessed the expression levels of *DKK1*, *YKT6*, and *HOXC6* genes in OSCC patients and compared them with expression levels in age- and gender-matched controls (Table 5). A two-sample t-test was used to check for statistical significance between cases and controls. No significant difference was found between cases and controls for any of the three genes.

**Table 5:** Mean expression levels of DKK1, YKT6, and HOXC6 genes in OSCC patients and controls Comparison between cases and controls was done using a two-sample t-test. Significance was considered at a p-value < 0.05. CI: confidence interval

Gene	Cases (n=12)		Controls (n=12)		t-value	p-value
	Mean	95% CI	Mean	95% CI		
DKK1	0.19372	-0.59867–0.98612	-0.00025	-0.60029–0.59978	-0.4295	0.6717
YKT6	-0.22171	-1.68297–1.23953	-0.00007	-1.06001–1.05985	0.2702	0.7895
HOXC6	-0.76128	-2.02418–0.50161	-0.00016	-1.14774–1.14741	0.9817	0.3369

The case population of our study was further subdivided into two groups: group I (cancer without any lymph node invasion) and group II (cancer with lymph node invasion and/or metastases). Statistical significance between the two groups for mean expression values of each of the three genes was assessed using a two-sample t-test. No statistical significance was found between the two groups (Table 6).

**Table 6:** Mean expression levels of DKK1, YKT6, and HOXC6 genes in group I and group II The two groups were compared using the two-sample t-test. Significance was considered at a p-value < 0.05. CI: confidence interval

Gene	Group I (n=6)		Group II (n=6)		t-value	p-value
	Mean	95% CI	Mean	95% CI		
DKK1	0.13010	-1.27090–1.53111	0.25735	-1.08242–1.59713	-0.1687	0.8694
YKT6	-0.22964	-3.31720–2.85791	-0.21379	-2.02547–1.59788	0.0977	0.9241
HOXC6	-0.70248	-3.25580–1.85083	-0.82007	-2.56471–0.92456	-0.0114	0.9911

## Discussion

This case-control study examined the expression levels of three genes (*DKK1*, *HOXC6*, and *YKT6*) in subjects with OSCC residing in central India and compared them with those of controls. A total of 240 subjects (120 OSCC patients and 120 age- and gender-matched controls) were recruited from the Department of Otorhinolaryngology at AIIMS Nagpur and included in the study. A questionnaire was administered to the subjects to determine each patient's demographic characteristics, after which whole blood was collected from each subject.

Serum was separated and stored at -80°C for the purpose of RT-PCR. For RT-PCR, RNA extraction was performed using Alchem BioPro kits, followed by cDNA synthesis. Using the primers designed for the three individual genes, Ct values were obtained for cases and controls of all three genes. Log-transformed fold change was calculated using the 2-∆∆CT method and used to measure differential expression between the two groups. For RT-PCR, 12 cases and 12 controls were analysed. The study also analysed the various risk factors commonly hypothesised to contribute to the development of OSCC. It was found that smoking, areca nut chewing, and alcohol consumption were major risk factors for the development of OSCC.

We also analysed the various risk factors and gene expression with respect to cancer severity by subdividing the case population of our study into two groups: group I (cancer without lymph node invasion) and group II (cancer with lymph node invasion and/or metastases).

Cases had an average age of 57.5 years (± 11.6), while controls had an average age of 58.7 years (± 9.2). There were 81 males (67.5%) and 39 females (32.5%) in both cases and controls. The cases and controls were chosen in an age- and gender-matched manner, so no statistical significance was observed between the groups.

Among modifiable risk factors, the number of subjects with a habit of areca nut chewing was significantly higher in cases than in controls. Cases had 5.33 times (95% CI: 2.99-9.49) greater odds of areca nut exposure than controls. Similarly, smoking addiction was also significantly more prevalent among cases, with cases having 5.5 times (95% CI: 3.16-9.56) higher odds of smoking compared to controls. Additionally, there was a significant difference in alcohol consumption habits, with cases having 9.85 times (95% CI: 5.46-17.74) greater odds of alcohol exposure than controls.

Mean expression levels of *DKK1*, *HOXC6*, and *YKT6* transcripts did not differ significantly between cases and controls. In the case group of our study population (n=12), 50% were classified as group I and 50% as group II. However, no significant difference in gene expression levels was found between the two groups for all three genes. The mean age of cases was 57.525 ± 11.639 years, which is comparable to the retrospective study by Ghai and Sharma (2022) [7] that assessed and compared the demographic profile of benign and malignant oral tumours in central India. The mean age of patients with malignant tumours was significantly higher than that of patients with benign tumours (51±14 versus 32±16 years; p<0.01).

According to the 2020 Global Cancer Statistics (GLOBOCAN) reported by Bray et al. (2021), head and neck squamous cell carcinoma (HNSCC) is more prevalent in males than females, with a male-to-female ratio of 2:1 [1]. The incidence of HNSCC also increases with age, with a mean age of 51 years. This was also observed in our study, where the mean age was 57.525 ± 11.639 years, and the incidence of OSCC was higher in males than in females, with a ratio of 2.07:1 (81 males and 39 females). The observed differences mainly arise from higher exposure of males to the concerned risk factors. Another reason might be that females are usually neglected with limited resources and, therefore, are an underdiagnosed population. Further studies may be undertaken to better quantify the burden of OSCC in the female population of central India.

Ghai and Sharma (2022) [7] reported that 23% of malignant tumours occurred in individuals aged 40 years or younger in the central Indian region (8). However, in our study, we found that only 11.67% of malignant tumours were in this age group. This discrepancy may be attributed to our study being conducted in a tertiary care hospital, which likely receives patients with more advanced stages of the disease and at older ages, following referrals from primary and secondary healthcare settings.

Gole et al. (2023) conducted a prospective cross-sectional study to assess the prevalence of oral cancer and its association with age, gender, type, site, and duration of habit [17]. The study found a male predominance in OSCC cases, with a male-to-female ratio of 2:1, further supporting our findings.

The prevalence of tobacco use is rising in developing countries due to increasing disposable income. In contrast, in developed nations, tobacco use has generally declined but has seen increases in certain demographics, particularly women [18,19]. Tobacco contains numerous carcinogenic substances, including polycyclic aromatic hydrocarbons, nitrosamines, aromatic amines, and aldehydes, which are released during high-temperature combustion. These chemicals can damage DNA in the oropharyngeal cells, contributing to cancer development.

According to Sadri et al. (2007), the combined odds ratio for tobacco smoking related to oral cancer was 4.65 (95% CI: 3.19-6.77) [20]. The highest combined odds ratio was observed in the Americas (OR = 7.65; 95% CI: 5.11-11.45), while the lowest was found in Asia (OR = 1.88; 95% CI: 0.95-3.71). In our study, we reported an odds ratio of 5.5 (95% C.I. 3.163-9.560).

Nocini et al. (2017) reported an odds ratio of 5.5‐fold (OR = 5.52; 95% CI: 2.57-9.80) in those smoking 70.8 packs per year, up to 7.3‐fold (OR = 7.27; 95% CI: 2.60-14.74) in those smoking 88.5 packs per year compared to nonsmoking people [21]. Although our study reported a similar odds ratio of 5.5, we did not have data on the pack-years smoked by individual subjects.

Chewing areca nut, or betel quid, is a major risk factor for oral and oropharyngeal HNSCC in South and Southeast Asia and Polynesia, accounting for more than half of cases [22]. This practice is often associated with cultural or recreational activities and produces psychoactive effects by antagonising GABA receptors, leading to increased alertness, euphoria, and appetite suppression.

Warnakulasuriya et al. (2020) conducted a case-control study with 304 oral cancer patients and 304 healthy controls, which reported an odds ratio of 5.4 (95% CI: 3.3-8.8) for areca nut chewers [23].

Madani et al. (2012) reported an odds ratio of 6.6 (95% CI: 3.0-14.8) in chewers compared to non-chewers in a case-control study conducted in India comprising 350 oral cancer cases and 350 controls [23].

Edirisinghe et al. (2023) conducted a case-control study of 105 patients with histologically confirmed primary OSCC and 210 age- and sex-matched controls in Sri Lanka [24]. Participants who had consumed alcohol at some point in their life had a 3.8-fold risk of developing OSCC (p=0.000). Those who consumed the locally made illicit liquor (Kasippu) had the greatest risk (OR = 8.45; p < 0.05) of developing OSCC when considering the type of alcohol consumed. Our study also reported a high odds ratio of 9.846 (95% CI: 5.464-17.744), considering the widespread use of locally made alcoholic drinks prevalent in this region. No such studies were conducted on the associated risk of alcohol consumption with the development of OSCC in the Indian population, to the best of our knowledge.

To our knowledge, this is the first study conducted to investigate the relative expression levels of the *DKK1*, *HOXC6*, and *YKT6* genes among OSCC subjects residing in central India.

Ogoshi et al. (2011) conducted a study that established the involvement of Dickkopf-1 (DKK1) in OSCC and its potential as a biomarker for early detection of lymph node metastasis [9].

Liu et al. (2023) conducted a study to investigate the role of the prognostic gene *DKK1* in OSCC. OSCC tissues were collected from patients who underwent surgical resection [25]. High *DKK1* expression is correlated with advanced tumour stage, lymph node metastasis, and poor prognosis. The study concluded that *DKK1* plays a crucial role in OSCC progression and may serve as a potential prognostic biomarker, but this could not be established in our study due to the limited sample size.

Chu et al. (2021) reported increased serum concentrations in various cancers, including gynaecological cancer, prostate cancer, hepatocellular carcinoma, bladder cancer, lung cancer, multiple myeloma, and osteosarcoma [26]. For HNSCC, a p-value of 0.159 was reported and was found to be not significant, which is in line with our study.

Hassan et al. (2006) examined the expression of 39 HOX genes in human oral tissues, hypothesising that aberrant expression might be linked to cancer development and progression [13]. They used RT-PCR to analyse these genes in 31 OSCC samples, 11 dysplasia samples, and 10 normal mucosa samples. They found that SCC cases with lymph node metastasis had higher *HOXC6* expression compared to those without metastasis; this association was not observed in our study.

Padam et al. (2022) examined various HOX gene signatures in oral cancer patients and found that *HOXA2* expression was elevated in dysplastic tissues but diminished during tumour progression [27]. *HOXB2* expression was reduced in both dysplastic and primary tumour samples. In contrast, *HOXA7*, *HOXA10*, *HOXB7*, *HOXC6*, *HOXC10*, *HOXD10*, and *HOXD11* were consistently upregulated in potentially malignant oral lesions as they advanced to oral cancer. Our study, however, did not observe a significant difference in *HOXC6* expression between cases and controls.

Yang et al. (2021) investigated the role of *YKT6* in OSCC prognosis and its potential role in immunotherapy response [14]. The study identified *YKT6* as a promising prognostic marker and predictor of immunotherapy response in OSCC, emphasising its significance in tumour progression and immune modulation.

Zhang et al. (2023) conducted a study utilising data from the Cancer Genome Atlas (TCGA), Gene Expression Omnibus (GEO) database, and bioinformatic tools to investigate the role of *YKT6* across 33 different tumour types [28]. They found that *YKT6* expression is significantly correlated with endothelial cells across various tumour types, including colon adenocarcinoma, HPV-positive HNSCC (HNSC-HPV+), ovarian cancer, rectal adenocarcinoma, and thyroid carcinoma. However, no statistical significance was observed in *YKT6* gene expression between cases and controls in our study.

However, the study faced certain limitations. Due to funding and time constraints, the full sample size could not be analysed, and only a limited number of samples were assessed for gene expression. Furthermore, serum samples were used in the absence of tissue collection equipment, which may have limited the accuracy of gene expression findings. Future research should focus on using tissue samples for a more precise evaluation and aim to correlate gene expression with immunohistochemistry findings to better understand gene involvement in cancer development and severity. This work lays the foundation for future studies that could lead to the development of targeted immunotherapies based on gene expression profiles in OSCC patients.

## Conclusions

Based on the results observed and analysed, the mean age for a cancer diagnosis was found to be around 58 years. The three genes under investigation, *DKK1*, *HOXC6*, and *YKT6*, were not significantly upregulated in either cases or controls, nor was any significant gene activity observed in relation to cancer severity. This study stands out as the first of its kind conducted on gene expression among OSCC patients in Central India, aiming to explore gene expression patterns for the potential development of biomarkers and immunotherapy targets. The study was strengthened by meticulous case and control selection, ensuring age- and gender-matching to minimise bias. Additionally, it identified major risk factors for OSCC in Central India, a region with the highest burden of this disease in the country.
